# Enhanced cognitive and perceptual processing: a computational basis for the musician advantage in speech learning

**DOI:** 10.3389/fpsyg.2015.00682

**Published:** 2015-05-21

**Authors:** Kirsten E. Smayda, Bharath Chandrasekaran, W. Todd Maddox

**Affiliations:** ^1^Department of Psychology, The University of Texas at AustinAustin, TX USA; ^2^Department of Communication Sciences and Disorders, The University of Texas at AustinAustin, TX USA

**Keywords:** plasticity, music, category learning, speech, OPERA

## Abstract

Long-term music training can positively impact speech processing. A recent framework developed to explain such cross-domain plasticity posits that music training-related advantages in speech processing are due to shared cognitive and perceptual processes between music and speech. Although perceptual and cognitive processing advantages due to music training have been independently demonstrated, to date no study has examined perceptual and cognitive processing within the context of a single task. The present study examines the impact of long-term music training on speech learning from a rigorous, computational perspective derived from signal detection theory. Our computational models provide independent estimates of cognitive and perceptual processing in native English-speaking musicians (*n* = 15, mean age = 25 years) and non-musicians (*n* = 15, mean age = 23 years) learning to categorize non-native lexical pitch patterns (Mandarin tones). Musicians outperformed non-musicians in this task. Model-based analyses suggested that musicians shifted from simple unidimensional decision strategies to more optimal multidimensional (MD) decision strategies sooner than non-musicians. In addition, musicians used optimal decisional strategies more often than non-musicians. However, musicians and non-musicians who used MD strategies showed no difference in performance. We estimated parameters that quantify the magnitude of perceptual variability along two dimensions that are critical for tone categorization: pitch height and pitch direction. Both musicians and non-musicians showed a decrease in perceptual variability along the pitch height dimension, but only musicians showed a significant reduction in perceptual variability along the pitch direction dimension. Notably, these advantages persisted during a generalization phase, when no feedback was provided. These results provide an insight into the mechanisms underlying the musician advantage observed in non-native speech learning.

## Introduction

Music training is a rich, multimodal experience that has been found to modify the brain in many positive ways. For instance, long-term music training is associated with enhanced processing of musical information such as pitch discrimination and perception (Schön et al., 2004; Tervaniemi et al., 2004; Magne et al., 2006; Bidelman et al., 2011; Zarate et al., 2012) rhythm production (Chen et al., 2008; Bailey et al., 2014), beat perception (Grahn and Rowe, 2012), and timbre discrimination (Crummer et al., 1994). Processing of musical information has also been studied in non-human primates. For instance, extensive pitch discrimination training has been used to characterize the plastic nature of the non-human auditory cortex (Brosch et al., 2004, 2005). In addition to musical information processing advantages, recent studies have also found that long-term music training is associated with advantages that extend beyond the musical domain, such as speech processing. For example, musicians show more robust neural encoding of speech sounds relative to non-musicians (Wong et al., 2007; Chandrasekaran et al., 2009; Kraus and Chandrasekaran, 2010) and outperform non-musicians in recognizing speech embedded in noise (Parbery-Clark et al., 2009; Strait and Kraus, 2011). Musicians also show superior non-native speech discrimination (Gottfried et al., 2004; Marie et al., 2011) and learning (Gottfried and Riester, 2000; Alexander et al., 2005; Wong and Perrachione, 2007; Lee and Hung, 2008) compared to non-musicians. While the musician advantage for learning non-native speech sounds is robust, the underlying mechanisms giving rise to this advantage are poorly understood.

Recently, a framework was developed to explore the mechanisms underlying the cross-domain auditory plasticity induced by long-term music training. The OPERA hypothesis posits that music training will affect the neural encoding of speech because: there is **O**verlap between the networks used to process both music and language; there is a greater **P**recision of processing of music relative to language; music elicits strong **E**motional experiences; **R**epetition is integral to music learning; and musical engagement requires sustained **A**ttention (Patel, 2011). The OPERA hypothesis was later updated to clarify the “precision” aspect of the hypothesis (Patel, 2012). More recently it was expanded to include the cognitive benefits of non-vocal music training on speech processing, motivation for using animal models, and preliminary data from a study investigating music training’s impact on speech perception in cochlear-implant users (Patel, 2014). Per this framework, music and speech share similarities in acoustics, such as pitch, timbre, and timing (Kempe et al., 2014), as well as higher-level cognitive processes such as working memory and attention (Besson et al., 2011; Kraus et al., 2012), suggesting that the musician advantage in learning non-native speech could arise from enhanced perceptual processing, cognitive processing, or both. To date, the evidence in support of these hypotheses comes from studies that target domain-general cognitive or perceptual processes with unique tasks. For instance, musicians show enhanced cognitive abilities compared to non-musicians in areas such as executive function (Bialystok and DePape, 2009), working memory (Parbery-Clark et al., 2009; Pallesen et al., 2010; George and Coch, 2011; Kraus et al., 2012; Strait et al., 2013), and switching (Hanna-Pladdy and MacKay, 2011), while a separate literature shows perceptual enhancements in speech processing (Parbery-Clark et al., 2011a,b, 2012; Zendel and Alain, 2012; White-Schwoch et al., 2013). To date, the cognitive and perceptual processes mediating the musician advantage in non-native speech learning has never been investigated within a single task. The current study addresses this shortcoming by examining non-native speech learning in musicians and non-musicians using traditional measures of performance (e.g., accuracy), and computational models that allow us to independently estimate the perceptual and cognitive processing.

We examine perceptual and cognitive processing within the specific theoretical framework of multidimensional (MD) signal detection theory (Ashby and Townsend, 1986; Maddox and Ashby, 1993). Within this framework, repeated presentations of the same physical stimulus yield unique perceptual effects that result in a multivariate normal distribution of perceptual effects (Green and Swets, 1967; Ashby and Townsend, 1986). Changes in the perceptual variances are associated with perceptual selectivity. To explore changes in perceptual processing as a function of musical training, we separately estimate a measure of perceptual selectivity (also referred to as perceptual variance or noise) along the pitch height and pitch direction dimensions. In addition, we can look at decision processes that involve constructing decision bounds (defined in detail later) that divide the perceptual space into separate response regions. Critically, perceptual and decisional processes are theoretically independent, and have unique, identifiable parameters (Green and Swets, 1967; Ashby and Townsend, 1986; Maddox and Ashby, 1993).

In the current study, we examine the extent to which long-term music training impacts learning to categorize Mandarin lexical pitch patterns. Mandarin Chinese is a tone language, wherein changes in pitch patterns within a syllable result in changes to word meaning. Learning to categorize the four pitch patterns in Mandarin is a challenging task for monolingual American adults (Wang et al., 1999), and therefore provides an excellent paradigm for studying the perceptual and cognitive mechanisms underlying learning. The four Mandarin Chinese tone categories and their descriptions are: T1, “high-level,” T2, “mid-rising,” T3, “low-dipping,” and T4, “high-falling” (Howie, 1976). Pitch height (how high or low a tone is) and pitch direction (average movement of a pitch) have been found to be the most prominent dimensions used in categorizing lexical tones such as in Mandarin (Gandour and Harshman, 1978; Gandour, 1983).

Native English speakers exhibit differential sensitivity to the dimensions underlying tone perception relative to native Mandarin Chinese speakers. MD scaling analyses of native English speakers and Mandarin speakers found that while English speakers weight the pitch height dimension equally to that of tone language speakers, they weight the pitch direction dimension less than tone language speakers (Gandour and Harshman, 1978; Chandrasekaran et al., 2007). This is likely due to the fact that pitch direction is not as salient a cue in English as it is in Mandarin, where it is required to distinguish pitch patterns that vary dynamically within the syllable. Although native English speakers and Mandarin speakers attend to the pitch height dimension to a similar degree, this dimension is highly influenced by variability in talkers (different talkers have different average pitches). In previous studies using the same computational modeling methods utilized in the current report, we have shown that the optimal decision strategy is one in which the participant attends to and utilizes both pitch height and pitch direction in making categorization judgments (Chandrasekaran et al., 2013; Maddox et al., 2013, 2014; Maddox and Chandrasekaran, 2014; Yi et al., 2014). This is referred to as a MD decision strategy and is contrasted with a unidimensional (UD) strategy in which the participant bases their decision solely on one stimulus dimension (usually pitch height). In the present study, we applied rigorous computational models to each participant’s response pattern on a block-by-block basis. We included one model that instantiates a MD strategy, two that instantiate UD strategies, and one that instantiates a random responder (RR) strategy. Computational models are necessary to make this distinction because the same accuracy rate can be obtained using qualitatively different strategies.

In addition to providing critical insights into the decisional strategies used by musicians and non-musicians, the computational models also allow us to explore perceptual processes independent of decisional processes. To explore changes in perceptual processing as a function of musical training, we separately estimate a measure of perceptual selectivity (also referred to as perceptual variance or noise) along the pitch height and pitch direction dimensions. Since pitch height is highly salient in English we make no strong predictions regarding the effects of musical training on perceptual selectivity along the pitch height dimension. However, although pitch direction is not as salient a feature in English as it is in Mandarin, musicians train for many hours a week to become sensitive to pitch direction (i.e., melodies), thus capitalizing on the narrow frequency tuning capabilities of the human primary auditory cortex (Bitterman et al., 2008). Therefore it is likely that musicians will show enhanced perceptual selectivity (i.e., reduced perceptual noise) along the pitch direction dimension compared to non-musicians. Detailed descriptions of the computational models can be found below in Section “Computational Modeling Descriptions.”

To summarize, we predict a musician advantage in non-native speech learning. Our goal is to go beyond accuracy measures and to provide mechanistic explanations for the musician advantage. We predict that this advantage is due to an increased use of optimal MD decision strategies, as well as enhanced perceptual selectivity along the pitch direction dimension.

## Materials and Methods

### Stimulus Characteristics

**FIGURE 1:**
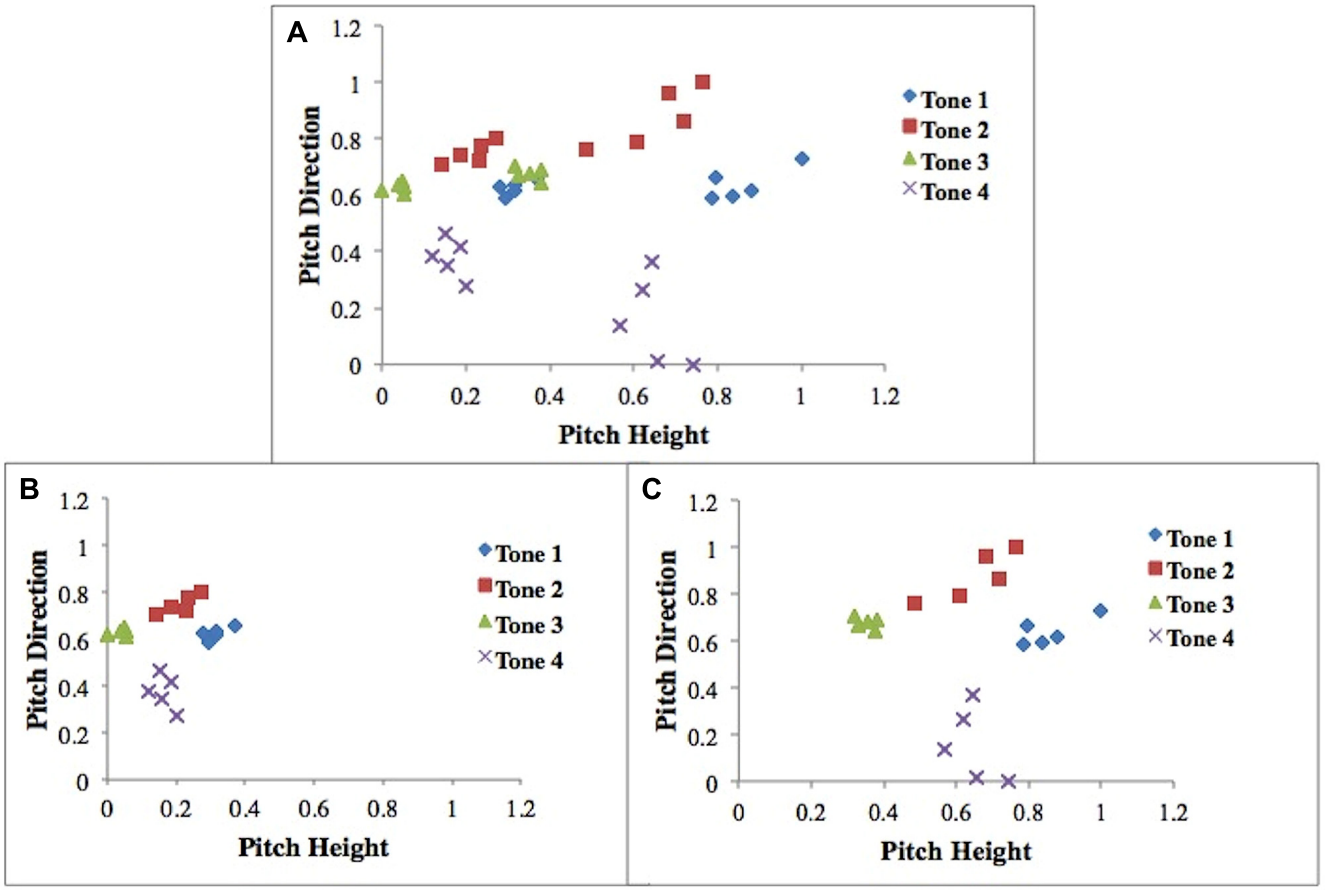
**Scatterplot of all stimuli (A).** Scatterplot of male-talker stimuli **(B)**. Scatterplot of female-talker stimuli **(C)**. Stimuli dimensions (Pitch Height and Pitch Direction) were normalized between 0 and 1. Pitch height refers to how high or low the pitch is, and pitch direction refers to (end pitch – start pitch)/duration.

Training stimuli consisted of the four Mandarin tones, tone 1 (T1), tone 2 (T2), tone 3 (T3), and tone 4 (T4) in the context of five syllables found in both Mandarin Chinese and English (“bu,” “di,” “lu,” “ma,” “mi”) by one male talker and one female talker (40 stimuli total). Both speakers are originally from Beijing, and stimuli were RMS amplitude and duration normalized (70 dB, 0.4 s) using the software Praat (Francis and Nusbaum, 2002; Wong et al., 2009; Perrachione et al., 2011). Five native speakers of Mandarin were asked to identify the tone categories (they were given four choices) and rate their quality and naturalness. High identification (>95%) was achieved across all five native speakers and speakers rated these stimuli as highly natural. We can represent these stimuli in a two-dimensional space with pitch height (how high or low a tone is) on the *x*-axis and pitch direction (average movement of the tone) on the *y*-axis (**Figure 1**). These two dimensions have been found to be especially relevant dimensions when categorizing the Mandarin tones (Francis et al., 2008).

### Participants

Fifteen musicians (7 female; mean age = 25 years, SD = 5.29) and fifteen non-musicians (12 female; mean age = 23 years, SD = 3.96) from The University of Texas at Austin and greater Austin, Texas community were paid $10 per hour for their participation. The University of Texas at Austin Institutional Review Board approved the study protocol, and informed consent was obtained from all participants. Exact thresholds were recorded for over half of the participants (8 of the 15 non-musicians; 9 of the 15 musicians). We conducted a mixed model ANOVA on the effect of ear (within subjects: left/right), frequency (within subjects: 500, 100, 2000 Hz), and group (between subjects: musician/non-musician) on pure tone audiometry thresholds. “Participant” was treated as a random variable. We found no difference between groups with respect to left and right ear thresholds [*F*(1,14) = 0.72, *p* = 0.41, partial η^2^= 0.05] or pure tone averages (500, 1000, 2000 Hz) [*F*(2,29) = 2.10, *p* = 0.14, partial η^2^= 0.13]. In addition participants reported no significant issues related to hearing. Musicians had at least 10 years of group or private instrumental lessons, and currently play or sing at least 3 h a week (instruments included organ, piano, flute, guitar, viola, and voice). Non-musicians had 3 years or less of group or private music lessons, and do not currently play an instrument. Participants’ musical history can be found in **Table 1**. Stimuli were presented at comfortable supra-threshold listening levels through Sennheiser HD 280 Pro headphones.

**Table 1:** Participants’ music history.

	Years of training	Age of onset, year	Hours play per week	Instrument
**Musician**				
1	15	7	20	Flute
2	15	8	28	Flute
3	11	5	6	Guitar
4	15	7	36	Organ
5	15	6	3	Piano
6	16	4	11	Piano
7	11	12	8.5	Piano
8	11	9	12	Piano
9	17	5	11	Piano
10	21	5	4	Piano
11	20	6	33	Piano
12	30	7	10	Viola
13	16	6	27	Viola
14	14	10	26	Voice
15	12	9	7	Voice
Mean	15.93	7.07	16.17	
**Non-musician**				
16	2	7	0	Flute
17	1	12	0	Flute
18	1	13	0	Guitar
19	1	9	0	Piano
20	2	8	0	Piano
21	3	8	0	Piano
22	0.5	10	0	Recorder
23	3	12	0	Saxophone
24	2	11	0	Trumpet
25	1	11	0	Violin
26	2	NA^∗^	0	Violin
27	0	NA	0	NA
28	0	NA	0	NA
29	0	NA	0	NA
30	0	NA	0	NA
Mean	1.23	10.10	0	


^∗^Subject 26 did not report age of onset.

### Procedure

On each trial, participants were presented with a single exemplar from one of four Mandarin tone categories (T1, T2, T3, or T4) and instructed to categorize the stimulus into one of four equally likely categories. During the training blocks, participants were given feedback after each trial and exposed to multiple talkers that were randomized within a block. Participants listened to 40 stimuli per block (4 tone categories × 5 syllables × 2 talkers). Each participant completed five 40-trial blocks of training and was instructed that high accuracy levels were possible. Participants generated a response by pressing one of four number button keys on the left side of the computer keyboard, labeled “1,” “2,” “3,” or “4.” Corrective feedback was provided for 1 s on the screen immediately following the button press and consisted of the word “Correct” or “No” followed by the label of the tone that was actually presented. For example, on a correct T1 trial the feedback display was as follows: “Correct, that was a category 1.” On an incorrect response trial where T4 was the correct response the feedback display was as follows: “No, that was a category 4.” A 1-s ITI followed the feedback.

After participants completed five 40-trial blocks, they completed one 20-trial generalization block. For the generalization block, all four tones and five syllables were presented, but were spoken by a different male speaker from the five training blocks. This resulted in 20 tokens (4 tones × 5 syllables × 1 new talker), and therefore 20 trials. In addition, feedback was not given. The generalization block was modeled separately from the five training blocks. The entire task lasted about 35 min.

#### Surveys and Neuropsychological Test

All participants completed a demographics survey, and a music and language experience survey. In addition, all participants completed WAIS-III Digit Span task to assess working memory capacity (Wechsler, 1997), and no difference was found between the two groups’ composite working memory sore (backward score + forward score) [*t*(28) = 1.51, *p* = 0.14]. Participants were matched on age and education (musicians: mean = 16.77 years, SD = 1.76; non-musicians: mean = 16.07, SD = 2.15).

### Computational Modeling Descriptions

#### Decisional Processing Assumptions

Accuracy rates provide an excellent source of information regarding how well an individual is performing in a task. Although a good starting point, one weakness of accuracy-based measures is that the same accuracy rate can often be achieved with qualitatively different strategies (e.g., UD or MD strategies). Within the domain of category learning, computational models can be utilized to address this shortcoming and can provide important insights into the nature of the strategy an individual is applying in a given task. In this study we apply a series of decision-bound models originally developed for application in the visual domain (Ashby and Maddox, 1993; Maddox and Ashby, 1993) and recently extended to the auditory domain by Maddox and Chandrasekaran (2014; Chandrasekaran et al., 2013; Maddox et al., 2013, 2014; Yi et al., 2014) on a block-by-block basis at the individual participant level because of problems with interpreting fits to aggregate data (Estes, 1956; Ashby et al., 1994; Maddox, 1999). We assume that the two-dimensional space (pitch height vs. pitch direction) displayed in **Figure 1A** accurately describes the perceptual representation of the stimuli, and based on the results from our earlier work (Maddox and Chandrasekaran, 2014), we also assume that participants applied category learning strategies separately to the male- and female-talker perceptual spaces (**Figures 1B,C**, respectively). Each model assumes that decision bounds (or category boundaries created by the participant as they learn the categories) were used to classify stimuli into each of the four Mandarin tone categories (T1, T2, T3, or T4).

**FIGURE 2:**
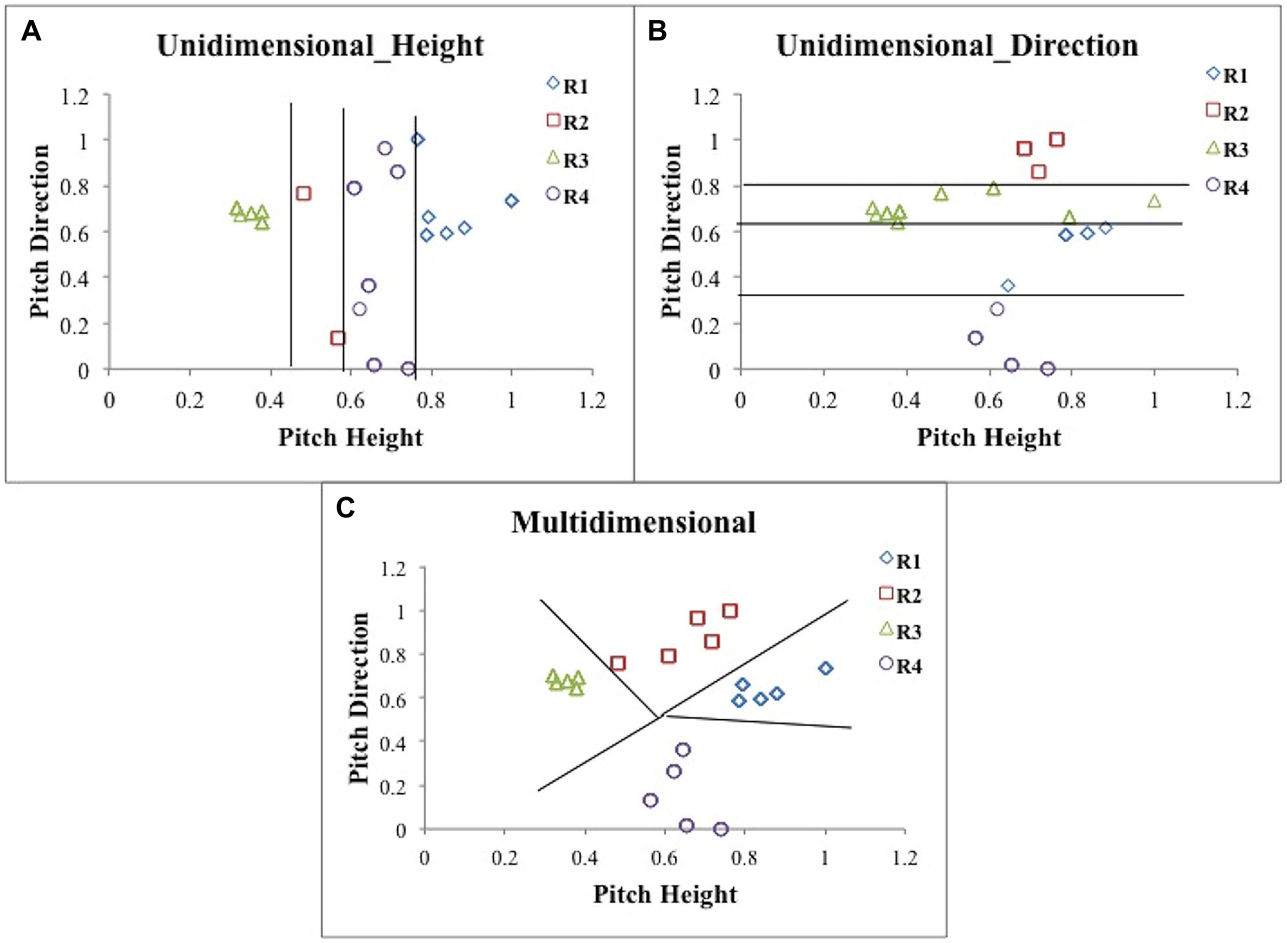
**Scatterplots of the responses along with the decision boundaries that separate response regions from a hypothetical participant using a version of the Unidimensional (UD)_Height (A), UD_Direction (B) and Multidimensional (MD; C) models as applied to the female talker stimuli shown in Figure 1C**.

To explore the types of strategies that participants used, we applied three types of models: UD, MD, and RR. **Figure 2** displays stimuli and response regions for the four tone categories generated from a hypothetical participant using strategies implicated by one version of the UD_Height model (**Figure 2A**), one version of the UD_Direction model (**Figure 2B**), and the MD model (**Figure 2C**). Each UD model assumed that the participant set three criteria along a given dimension, which effectively partitioned the perceptual space into four response regions. For example, the UD_Height model assumes that the participant sets three criteria along the pitch height dimension, which are used to separate the stimuli into those that are low, medium–low, medium–high, or high pitch height. Importantly, this model ignores the pitch direction dimension. The eight most reasonable variants of the model were examined and differ only in the assignment of the category labels (T1, T2, T3, T4) to response regions (low, medium-low, medium–high and high, respectively). Therefore, the eight most reasonable variants were: 3214, 3412, 3241 (shown in **Figure 2A**), 3421, 2314, 4312, 2341, and 4321. For example, a participant who carved up the space using the 3241 variant of the model would categorize a low tone as category 3, a medium–low tone as category 2, a medium–high tone as category 4, and a high tone as category 1. The UD_Direction model assumes that the participant sets three criteria along the pitch direction dimension. The model assumes that the three criteria along the pitch direction dimension are used to separate the stimuli into those that have a low slope, medium–low slope, medium–high slope, or high slope. Importantly, this model ignores the pitch height dimension. The two most reasonable variants of the model were examined and differ only in the assignment of category labels (T1, T2, T3, T4) to response regions (low, medium–low, medium–high, and high slopes). These were: 4312 and 4132 (shown in **Figure 2B**). Each UD model contains three free decision parameters—three criteria along the relevant dimension.

The MD model that we used also partitioned the space into four separate response regions, but unlike the UD models, the MD model focused on both the pitch height and pitch direction dimensions. In addition, whereas the UD model decision bounds were vertically oriented (in the UD_Height model) or were horizontally oriented (in the UD_Direction model), in the MD model the decision bound orientations were not constrained. A model of this sort can be instantiated in a number of ways. In line with some of our previous work (Maddox et al., 2013, 2014; Maddox and Chandrasekaran, 2014; Yi et al., 2014), we used a simple-prototype model framework in which each category is represented by a single prototype and each exemplar is classified into the category with the most similar prototype. Because the location of one of the prototypes can be fixed, and since a uniform expansion or contraction of the space will not affect the location of the resulting response region partitions, the MD model contains five free decision parameters that determine the location of the prototypes, and a single free parameter that represents noise in their placement. **Figure 2C** displays a scatterplot of the stimuli and response regions for the four tone categories generated from a hypothetical participant using one version of the MD model. A key feature of this model is that it assumes the participant is integrating information from both pitch height and pitch direction dimensions in their classification of Mandarin tones, making this a model that implicates a MD strategy. Importantly, we introduce the decisional models we present here, and the perceptual models we present in Section “Perceptual Processing Assumptions” as “cognitive” and “perceptual” models within a specific theoretical framework – multiple signal detection theory (Ashby and Townsend, 1986; Maddox and Ashby, 1993). These models are referred to as “cognitive” models because working memory, attention, and executive functioning are relevant to the distinction between UD and MD strategies. We explore working memory capacities of UD and MD strategy users in section Working memory and cognitive strategies.

The third model is a RR model that assumes that the participant guesses on each trial.

#### Perceptual Processing Assumptions

Whereas **Figures 1A–C** denotes the mean perceptual effects of the stimuli, variability in the trial-by-trial perceptual effects is estimated from the data. We assume that the perceptual variance along the pitch height dimension is identical across all 40 stimuli and that the perceptual variance along the pitch direction dimension is identical across all 40 stimuli (referred to as a stimulus invariant perceptual representation; Ashby and Maddox, 1992; Maddox, 2001, 2002; Maddox and Dodd, 2003), but that the perceptual variance along the pitch height and pitch direction dimensions are uncorrelated (referred to as perceptual independence; Ashby and Townsend, 1986; Ashby, 1988). In other words, while we estimate the perceptual variability along the pitch height dimension separately from that along the pitch direction dimension, we assume those variability estimates are constant across stimuli (stimulus invariance), and that the perceptual covariance between pitch height and pitch direction is zero (perceptual independence). A smaller perceptual variance is associated with a more veridical percept. The decisional processes introduced above, and the perceptual processes introduced in this section are independent of one another (Green and Swets, 1967; Maddox and Ashby, 1993).

#### Model Fitting Procedure

In this section, we elaborate on the procedures used to fit models to behavioral data. On each trial, the participant is presented with a single stimulus and emits one categorization response. Thus for each stimulus the observed probability of responding T1–T4 is either 1 or 0 with three of these responses having an observed probability of 0 and one of 1. For example, if the participant generated a T1 response on trial 1, then the observed probability of responding T1, T2, T3, and T4 would be 1, 0, 0, and 0, respectively. The same holds for each of the 40 trials in a block. For a given model and a fixed set of parameters, the model generates a set of predicted response probabilities for each of the 40 trials. The observed and predicted values are combined using maximum likelihood, and are used to produce an Akaike information criterion (AIC; Akaike, 1974) value:

(1) $$AIC_{i}\,\;=\;-2lnL_{i}\,\;+\;2V_{i}$$

where *L*_i_ is the maximum likelihood for model *i*, and *V*_i_ is the number of free parameters in the model. The model parameters are adjusted until the smallest AIC value is identified, and this is defined as the best fitting version of that model for a given set of data. This process is repeated for all of the models and the model with the smallest AIC value is defined as the best fitting model for that data set. Notice that AIC penalizes models with more free parameters. Thus, if two models provide equivalent maximum likelihood fits to a set of data, but one has more free parameters, the model with more free parameters will be rejected in favor of the model with fewer free parameters.

### Data Analysis

Several of our results derive from an examination of the effects of music training on performance across blocks of trials, such as accuracy, and perceptual selectivity measures from the computational models. In these cases, we conducted a 2 between group (musician vs. non-musician) × 5 within group (block: 1–5, repeated measure) mixed design ANOVA with “participant” as a random variable. Other results derive from simple comparisons between musician and non-musicians. These include the first block of trials best fit by a MD strategy model, total number of blocks fit by a MD strategy model, working memory comparisons between MD and UD users, and measures of accuracy and perceptual variance in the generalization block. For these analyses, we used *t*-tests to compare measures between groups. All analyses were carried out using R version 3.0.3 (R Core Team, 2014).

## Results

We first present accuracy analyses comparing block-by-block training and generalization performance between musicians and non-musicians. Then we present model-based analyses to explore the types of decision strategies that participants use to learn during the task, working memory comparisons of different strategy users, and the magnitude of perceptual noise along the pitch height and pitch direction dimensions.

### Accuracy Results

**FIGURE 3:**
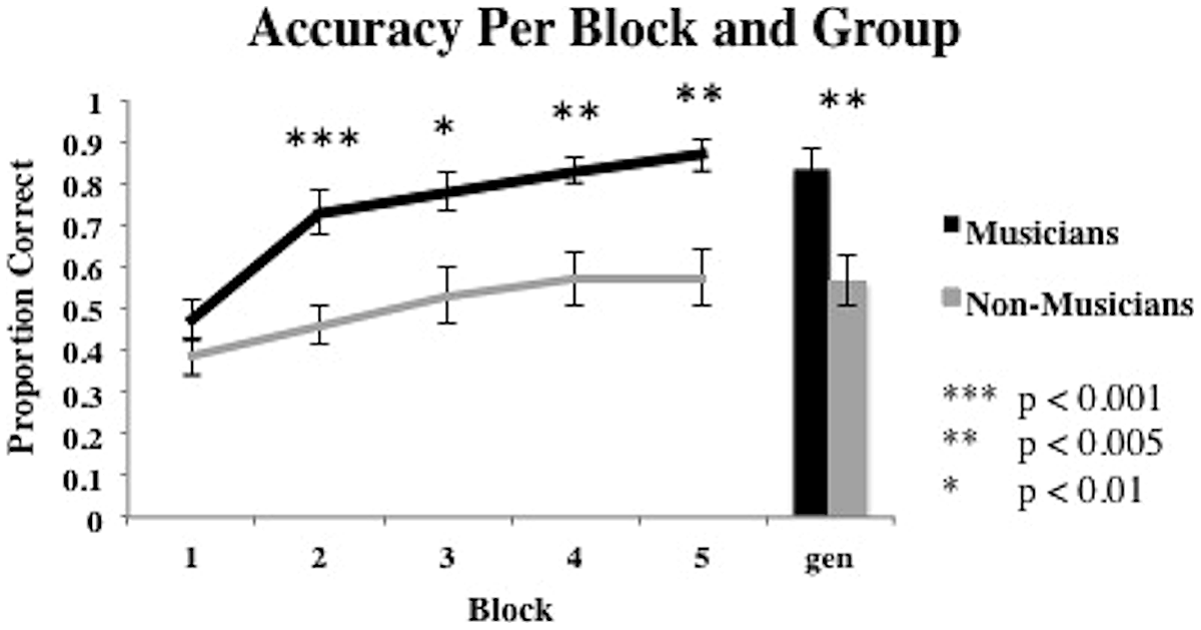
**Average proportion correct for the five training blocks and generalization block for musicians and non-musicians.** Bars represent SEM.

Learning curves for the musicians and non-musicians are presented in **Figure 3**. We begin with a 2 group (between subjects: musician vs. non-musician) × 5 training block (within subjects: blocks 1–5) mixed design ANOVA on the accuracy data with “participant” as a random variable. The main effect of participant group was significant [*F*(1,28) = 11.07, *p* = 0.0018, partial η^2^= 0.3] and suggests a performance advantage for musicians (average accuracy = 0.74) over non-musicians (average accuracy = 0.50). The main effect of block was also significant [*F*(4,112) = 47.60, *p* < 0.001, partial η^2^ = 0.063]. Finally, the interaction between participant group and block was significant [*F*(4,112) = 5.911, *p* < 0.001, partial η^2^= 0.174]. *Post hoc* pairwise comparisons of the groups at each block suggest that the musician advantage held in all blocks except block 1 (all *p*’s < 0.01). In addition, we tested the difference in learning trajectories between the two groups by conducting polynomial contrast tests on accuracy between the musician and non-musician groups across blocks. Results revealed a significant linear relationship of the group × block interaction [*F*(1,112) = 14.01, *p* < 0.001, partial η^2^= 0.111], a significant quadratic trend of the interaction [*F*(1,112) = 4.25, *p* < 0.05, partial η^2^= 0.037], and a significant cubic trend of the interaction [*F*(1,112) = 4.59, *p* < 0.05, partial η^2^= 0.039]. Contrast analyses using the linear, quadratic, and cubic scores for each participant indicated that the linear trend was significantly different for the musician and non-musician groups. The average linear increase in accuracy for the musician group (*M* = 0.49, SD = 0.41) is significantly larger than the average linear increase in accuracy for the non-musician group [*M* = 0.89, SD = 0.42; *t*(148) = 5.93, *p* < 0.001]. The quadratic trend also differed significantly for the musician and non-musician groups across blocks and was significantly greater for the non-musician group (*M* = -0.17, SD = 0.27) than for the musician group (*M* = -0.43, SD = 0.29) [*t*(148) = 5.93, *p* < 0.001]. Lastly, the cubic trend was significantly different for musicians and non-musicians across blocks. The cubic trend from the musicians was significantly larger for musicians (*M* = 0.20, SD = 0.24), than non-musicians [*M* = -0.04, SD = 0.21) [*t*(148) = 6.34, *p* < 0.001]. These results suggest different learning trajectories for musicians and non-musicians, where across blocks, musicians show a significantly stronger linear and cubic trend relative to non-musicians, who show a significantly stronger quadratic trend. As suggested by an examination of **Figure 3**, generalization performance for musicians was superior to that for non-musicians [*t*(28) = 3.48, *p* < 0.005].

To determine whether more training trials might result in a different pattern of accuracy rates for musicians and non-musicians, we compared accuracies in block 4 and 5 for musicians and non-musicians separately. Using two one-way repeated measures ANOVA’s, results reveal that accuracy rates for both musicians and non-musicians did not significantly change from block 4 to 5 [musicians: *F*(1,14) = 2.88, *p*= 0.11; non-musicians: *F*(1,14) = 0.01, *p* = 0.91].

Taken together, these data suggest that musicians show better Mandarin tone category learning and generalization than non-musicians. These findings replicate a large body of work in showing an accuracy advantage in learning non-native speech categories for musicians relative to non-musicians (Gottfried and Riester, 2000; Alexander et al., 2005; Wong and Perrachione, 2007; Lee and Hung, 2008). Next we explore computational modeling of participants’ responses to better understand the locus of the musician performance advantage.

### Computational Modeling Results

The accuracy-based analyses suggest that musicians showed a learning and generalization advantage over non-musicians when asked to categorize Mandarin tones. Accuracy measures are informative, but they do not provide a mechanistic explanation for this performance advantage – for instance, whether this advantage is due to cognitive and/or perceptual processing advantages in musicians. It is possible that non-musicians are using the same strategies as musicians, just sub-optimally, or they could be using different strategies altogether. In addition, musicians and non-musicians may show similarities or differences in perceptual selectivity along each dimension. Model-based analyses allow us to address these important questions.

#### Cognitive Strategies and Accuracy Rates across Blocks

**FIGURE 4:**
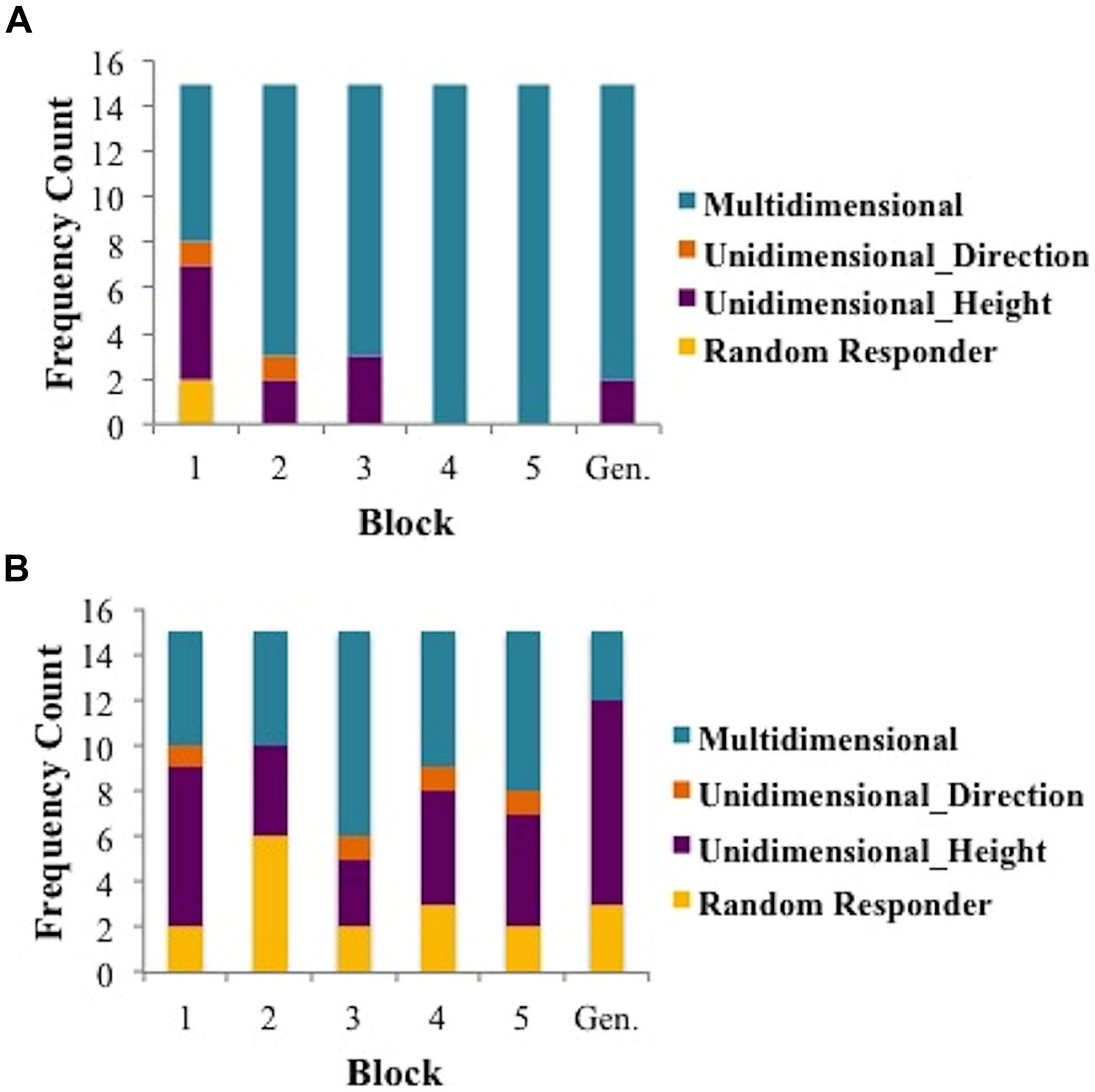
**Strategy use counts per block for musicians (A) and non-musicians (B)**.

In this section, we compare the cognitive strategies used by musicians and non-musicians during Mandarin tone category learning. Specifically, we compare the use of a MD, UD, and RR strategies across musicians and non-musicians. A breakdown of strategies per block and group can be found in **Figure 4**.

To investigate the use of strategies over the course of the five training blocks, we examined three aspects of the data between musicians and non-musicians. First we determined the first block of trials for which the MD model provided the best account of the data and compared these values for musicians and non-musicians. Second, we determined the total number of blocks of trials for each participant for which the MD model provided the best account of the data and compared these values for musicians and non-musicians. Finally, we examined the learning curves for musicians and non-musicians whose final block of data was best fit by either a MD or a UD strategy. To determine the first block of trials for which musicians (as a group) and non-musicians (as a group) used a MD strategy, we identified the first block of trials for each participant for which the MD model provided the best account of the data. We then computed the average of these blocks for musicians and non-musicians separately. For instance, if the first block of trials for which a MD strategy best fit the data from musicians 1–3 were blocks 3, 4, and 4, then the average block when they first used a MD strategy would be block 3.67. We found that the first use of a MD strategy occurred significantly earlier for musicians (average 1.87 blocks) than for non-musicians (average 3.20 blocks) [*t*(28) = 2.24, *p* < 0.05]. Next, we examined the number of blocks of trials for which a MD strategy provided the best fit to the data for musicians and non-musicians. We found that the number of blocks of trials best fit by a MD model was larger for musicians (average 4.07 blocks) than non-musicians (average 2.13 blocks) [*t*(28) = 3.24, *p* < 0.01].

**FIGURE 5:**
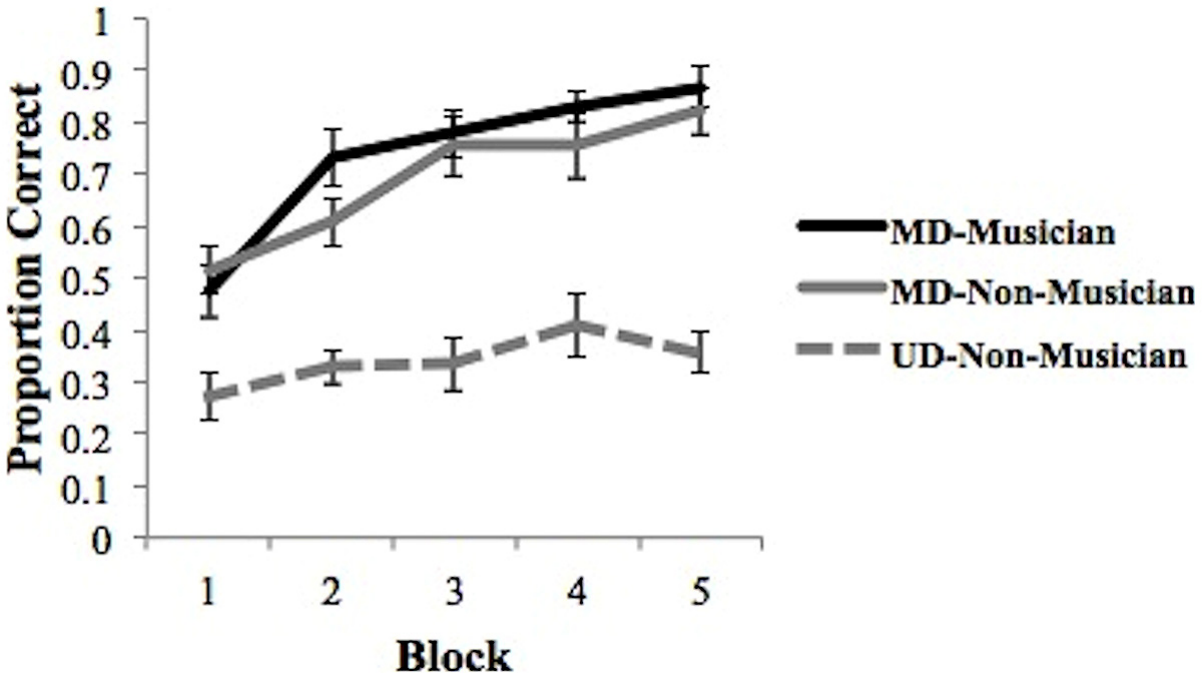
**Average proportion correct across all training blocks for MD musicians, MD and UD non-musicians based on final block strategy.** Bars represent SEM.

Finally, we examined the learning curves associated the best fitting model during the final training block. We classified participants as UD-Musician, UD-Non-Musician (UD groups also included those best fit by RRs), MD-Musician, and MD-Non-Musician based upon the best fitting model from block five. As suggested by an examination of **Figure 4**, none of the 15 musicians’ data was best fit by a UD model in block 5. Thus, we cannot generate a learning curve for this group. The goal of this analysis was to determine how the strategy used in the final block of trials might affect the course of learning. **Figure 5** shows the learning curves for each group based on this classification. A 3 group (between subjects: musicians using MD, non-musicians using MD, non-musicians using UD, or RR strategies) × 5 training block (within subjects) mixed design ANOVA conducted on proportion correct (accuracy) revealed a significant main effect of group [*F*(2,27) = 23.69, *p* < 0.0001, partial η^2^ = 0.64], a significant main effect of block [*F*(4,108) = 52.99, *p* < 0.0001, partial η^2^= 0.66], and a significant interaction between block and group [*F*(8,108) = 5.38, *p* < 0.0001, partial η^2^ = 0.28]. *Post hoc* pair-wise comparisons with Bonferroni correction of the group main effect revealed that both musicians and non-musicians using MD strategies were significantly more accurate than non-musicians using UD strategies in all blocks (all *p*’s < 0.01). The comparison of musicians and non-musicians who used MD strategies did not reach significance (*p* > 0.38). Thus, although musicians are more likely to utilize MD strategies than non-musicians, those musicians and non-musicians who use MD strategies do so with nearly the same accuracy. This is an important finding as it suggests a critical mechanism (MD strategy use) associated with enhanced speech learning (Chandrasekaran et al., 2013; Maddox et al., 2013, 2014; Maddox and Chandrasekaran, 2014; Yi et al., 2014).

#### Working Memory and Cognitive Strategies

We also investigated any working memory differences between participants who arrived at a UD strategy versus participants who arrived at a MD strategy in block 5. Importantly, we did not find any working memory difference between our musician group and non-musician group [*t*(28) = 1.51, *p* = 0.14]. While this does not replicate previous work (Parbery-Clark et al., 2009; Pallesen et al., 2010; George and Coch, 2011; Kraus et al., 2012; Strait et al., 2013), our computational modeling can give us insight into why this may be.

Executive function is critical for MD strategy use as it is a complex decisional process requiring the maintenance of multiple pieces of auditory information in order to make a categorical judgment. Thus, we predict that participants who use MD strategies will have a higher working memory capacity. To test this, we conducted a one-way ANOVA of group (between subjects: musician, non-musician) and block 5 strategy [between subjects: MD, non-MD (UD and RR)] on composite working memory scores (forward score + backward score). The ANOVA revealed a significant main effect of strategy [*F*(1,27) = 7.28, *p* < 0.01], but no significant main effect of group [*F*(1,27) = 2.80, *p* = 0.11] on composite working memory score. *Post hoc t*-tests between groups suggest that block 5 MD users have a significantly higher working memory composite score than block 5 non-MD users [*t*(28) = 3.21, *p* < 0.005]. Within just non-musicians, block 5 MD users have a significantly higher working memory composite score relative to block 5 non-MD users [*t*(13) = 2.55, *p* < 0.05]. In addition, there is no difference in working memory composite scores between non-musician block 5 MD users and musician block 5 MD users [*t*(20) = 0.27, *p* = 0.79]. Because there were no UD or RR musicians, we could not compare their working memory scores to those of MD musicians. These results suggest that working memory abilities may partially explain who uses a MD strategy by the end of training, regardless of music training.

#### Strategies and Accuracy Rates in Generalization Block

Turning to the generalization block, a Fisher exact test reveals that there were significantly more musicians using a MD strategy relative to non-musicians using a MD strategy (*p* < 0.001). Next, we explored the accuracy rates associated with musicians and non-musicians who were either MD strategy users or UD strategy users in the generalization block (strategy counts in **Figure 4**) and found that non-musicians using MD strategies obtained marginally higher accuracy rates than non-musicians using UD strategies [*t*(10) = 2.03, *p* = 0.07]. Likewise, musicians using MD strategies obtained significantly higher accuracy rates than musicians using UD strategies [*t*(13) = 2.43, *p* < 0.05] whereas musicians using MD strategies were no more accurate than non-musicians using MD strategies [*t*(14) = 0.59, *p* = 0.56]. Just as in the training blocks, these results suggest that employing a MD strategy, regardless of music experience, enhances accuracy. However, these results should be interpreted with caution due to the small sample sizes.

#### Computational Modeling Results of Perceptual Representation Across blocks

In this section, we examine the effects of musical training on perceptual selectivity along the pitch height and pitch direction dimensions. A number of studies in the literature (Goldstone, 1994; Maddox and Bogdanov, 2000; Maddox, 2001, 2002; Maddox and Dodd, 2003) suggest that perceptual forms of selectivity often follow when decisional forms of selectivity are operative, but not always (Filoteo and Maddox, 1999). Given that English speakers naturally weight pitch height, due to its relevance in English, it is reasonable to suppose that musicians and non-musicians will not show any differences in perceptual selectivity along the pitch height dimension before training (however, see Perrachione et al., 2013 regarding the influence of music experience on perceptual selectivity at the sentence-level). It is likely, however, that musical training leads to enhanced perceptual selectivity along the pitch direction dimension and thus musicians will show smaller estimates of perceptual noise. Because we focus on the perceptual variability estimates, we wanted to use the model that best accounted for the data. This, by definition, is the most general MD model.

**FIGURE 6:**
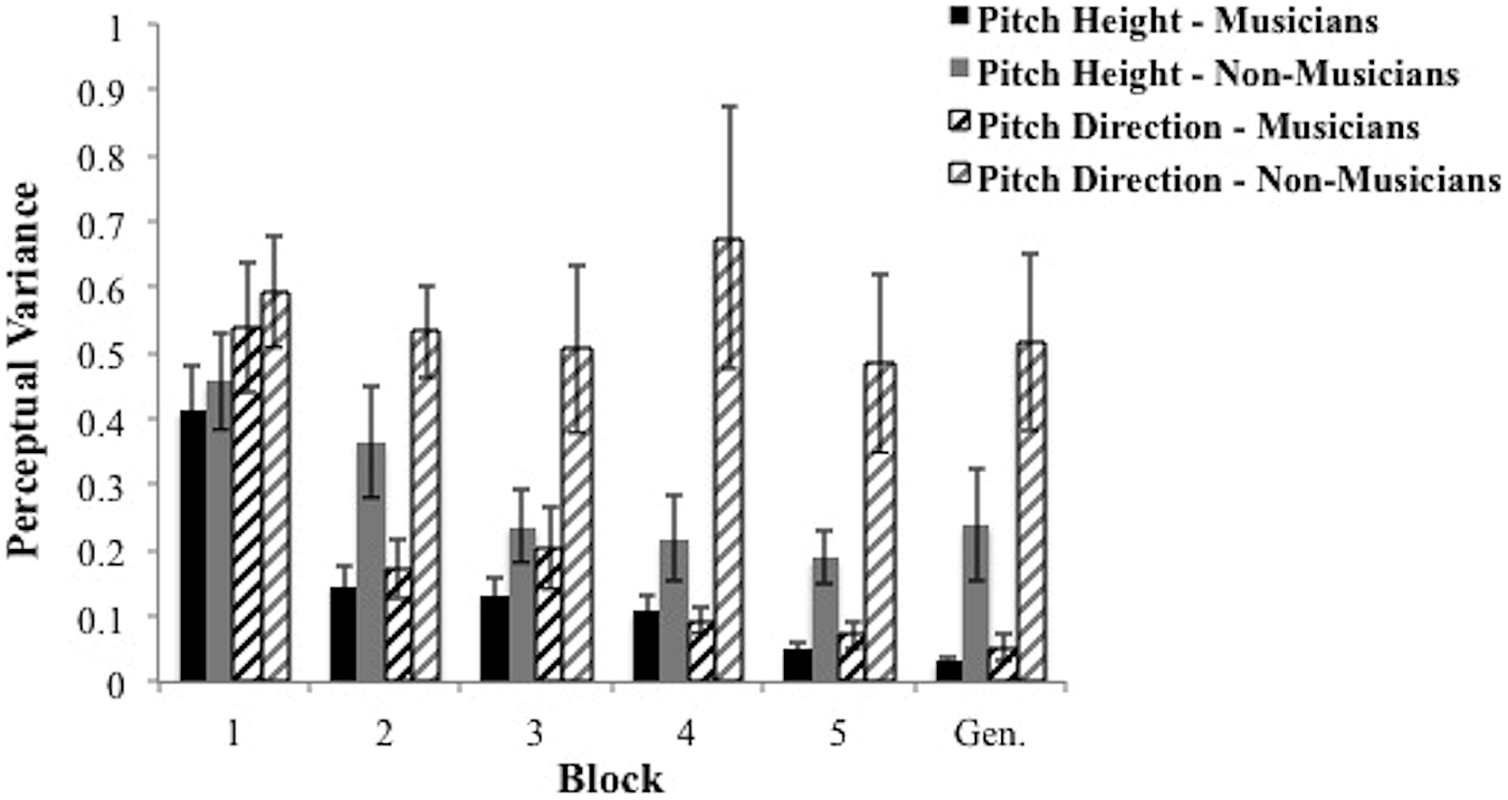
**Average perceptual variance of groups across training blocks and generalization block in the pitch height and pitch direction dimensions. Bars represent SEM**.

First, we examined the effects of musical training on perceptual selectivity along the pitch height dimension. We conducted a 2 group (between subjects) × 5 block (within subjects) mixed design ANOVA, with “participant” as a random variable. We found a main effect of group [*F*(1,28) = 4.16, *p* = 0.051, partial η^2^ = 0.129], and a main effect of block [*F*(4,112) = 23.59, *p* < 0.001, partial η^2^ = 0.457], but no interaction [*F*(4,112) = 1.55, *p*= 0.194, partial η^2^ = 0.052]. Musicians showed better perceptual selectivity in the form of smaller perceptual variance (mean = 0.17) compared to non-musicians (mean = 0.29). In addition, perceptual variance across groups decreased with learning (mean of block 1 = 0.43; mean of block 5 = 0.12). These results are displayed in **Figure 6**.

Second, we examined the effects of musical training on perceptual selectivity along the pitch direction dimension. We conducted a 2 group (between subjects) × 5 block (within subjects) mixed design ANOVA, with “participant” as a random variable. We found a significant interaction [*F*(4,112) = 2.87, *p* < 0.05, partial η^2^= 0.093], along with a significant main effect of group [*F*(1,28) = 11.38, *p* < 0.005, partial η^2^= 0.289], and a significant main effect of block [*F*(4,112) = 3.62, *p* < 0.01, partial η^2^= 0.115]. To identify the locus of the significant interaction, we conducted two analyses. First, we ran *t*-tests comparing musicians and non-musicians at each block. We found significant smaller perceptual variance estimates for musicians in all blocks except the first [block 1: *t*(28) = 0.42, *p* = 0.68; block 2: *t*(28) = 4.33, *p* < 0.0005; block 3: *t*(28) = 2.13, *p* < 0.05; block 4: *t*(28) = 2.92, *p* < 0.01; block 5: *t*(28) = 3.01, *p* < 0.01]. Next, we conducted separate one-way repeated measures ANOVA’s within each group and found musicians’ perceptual variance estimates along the pitch direction dimension declined significantly across blocks [*F*(4,56) = 15.24, *p* < 0.0001, partial η^2^= 0.521] whereas non-musicians’ did not [*F*(4,56) = 0.57, *p* = 0.69, partial η^2^= 0.039].

#### Computational Modeling of Perceptual Representation in Generalization Block

Here we examine the perceptual variance estimates in the generalization block. These analyses allow us to determine how perceptual variability along the pitch height and pitch direction dimensions changes in the context of a new talker and no feedback. Perceptual variance estimates were smaller for musicians relative to non-musicians along both the pitch height [*t*(28) = 2.42, *p* < 0.05], and pitch direction dimensions [*t*(28) = 3.39, *p* < 0.005]. These results are depicted in **Figure 6**. We also compared the perceptual variance estimates in the final training block to those in the generalization block. Interestingly, the pitch height and pitch direction perceptual variance estimates were numerically smaller in the generalization block than in the final training block for musicians, but were numerically larger for non-musicians. Even so, the only one of these comparisons to reach statistical significance was for musicians along the pitch height dimension [*t*(14) = 2.21, *p* < 0.05].

## Discussion

We examined the effects of long-term musical training on non-native speech learning in native English speakers, none of whom had prior experience with Mandarin tones. Our results show a musician advantage (average accuracy = 0.74) relative to non-musicians (average accuracy = 0.50) in learning to categorize naturally produced Mandarin tones. Our results are consistent with previous studies that have identified a musician advantage in learning speech categories (Gottfried and Riester, 2000; Alexander et al., 2005; Wong and Perrachione, 2007; Lee and Hung, 2008). While accuracy differences help identify a cross-domain (between music and speech learning) advantage for musicians, they do not provide information on the specific mechanistic underpinnings of the advantage. To this end, we employed computational modeling analyses to examine the locus of the musician advantage. Specifically, our models specified decisional strategies used by musicians and non-musicians, as well as perceptual processes that are independent of the decisional processes. The computational modeling results revealed that musicians used the optimal, MD strategy faster, and more frequently than non-musicians. This suggests musicians have enhanced cognitive processing supporting categorical decisional judgements relative to non-musicians as a group.

Importantly, the model-based analyses allow us to examine decision processes in each individual. Although musicians used MD strategies faster and more frequently than non-musicians, when compared to non-musicians who used MD strategies by block 5, there were no differences in accuracy rates. In addition, across participant groups, participants who used MD strategies in the final training block had a significantly higher working memory composite score than those who used UD strategies. Specifically, musicians and non-musicians who used MD strategies in block 5 were no different in their composite working memory scores. In addition, non-musicians who used MD strategies in block 5 had a significantly higher working memory score than non-musicians who had did not use a MD strategy in block 5. These are critical findings as they suggest a mechanism for the musician advantage; namely, an increased use of MD strategies, since musicians and non-musicians who used MD strategies by the end of the training were very similar with respect to accuracy and working memory capacity.

Increased use of MD strategies leads to enhanced speech learning, but changes in perceptual processing may also explain better performance. Importantly, these parameters are theoretically independent from the decision parameters (Green and Swets, 1967; Maddox and Ashby, 1993) and in some cases are empirically independent (Filoteo and Maddox, 1999). The current results suggest that both musicians and non-musicians show increased perceptual selectivity (i.e., reduced perceptual variance or noise) along the pitch height dimension with learning. However, only musicians show increased perceptual selectivity (or more veridical perception) along the under-weighted pitch direction dimension. Together, this suggests that the performance advantage in learning non-native speech sounds for musicians relative to non-musicians is due not only to cognitive processes, but also perceptual processes and is consistent with enhanced perceptual representation of dynamic pitch changes in musicians, relative to non-musicians (Wong et al., 2007).

Why would long-term music training promote cross-domain auditory plasticity? Studies examining plasticity related to music training have examined basic perceptual encoding of auditory signals as well as higher-level linguistic and cognitive processes. For example, musicians show enhanced encoding of linguitic pitch patterns at the level of the midbrain/brainstem relative to non-musicians (Wong et al., 2007). Such perceptual encoding advantages could lead to faster speech learning in musicians by relaying a more faithful representation of the speech signal to the cortex than non-musicians. A general cognitive/decisional advantage could drive enhanced speech learning as well. In fact, a previous proposal posits a reciprocal process where cognitive advantages drive perceptual advantages in a top–down manner (Strait et al., 2010). The OPERA hypothesis suggests that music training places a significantly greater demand on the perceptual and/or cognitive circuitry that is shared between music and speech (Patel, 2014). In addition, recent findings suggest common mechanisms underlying music aptitude and speech-sound processing (Kempe et al., 2014). Thus, long-term training could alter cognitive and perceptual processes that are common to music and speech, resulting in enhanced learning of components shared between the two domains.

In the current study we examined the extent to which music training enhanced learning of non-native, linguistic pitch patterns. Pitch is a basic element in music and speech, and both domains use pitch patterns to convey information extensively. In English speech, pitch patterns can convey information related to prosody and emotion. Pitch patterns are also used in some languages (e.g., Mandarin Chinese) within a syllable to change word meaning. Native English-speaking adults struggle in learning Mandarin pitch patterns and often confuse one tone category with another (Wang et al., 1999; Chandrasekaran et al., 2010). Our results show that music training can enhance the ability to categorize non-native liguistic pitch patterns. Computational modeling helps pinpoint the locus of this advantage by showing that musicians use the optimal MD strategy sooner and more often than non-musicians. In addition, musicians shower greater perceptual selectivity of the stimuli along the pitch direction dimension relative to non-musicians.

Lexical tones are well characterized by a MD space with two dimensions related to pitch (pitch height and direction) that can help disambiguate tone categories. The relative weighting between dimensions is language-dependent, where native English speakers tend to weight pitch direction less than native Mandarin speakers, reflecting the relative difference in the use of this dimension in their language (Gandour and Harshman, 1978; Chandrasekaran et al., 2007). Thus, native English speakers focus predominantly on pitch height to disambiguate tone categories. In previous studies using computational models we found that relying on only one of the two dimensions during learning (a UD decision strategy) is a sub-optimal strategy (Maddox et al., 2013; Yi et al., 2014). For example, an over-reliance on pitch height (is it high or low?) is not optimal because it leads to confusions between the rising and the falling tones (which have similar average heights but differ considerably in direction). Pitch height is also highly talker-dependent; for example, it is a critical cue in differentiating male and female talkers. Thus, an over-reliance on this dimension may lead to category confusions across talkers. The computational modeling results of the current study show that relative to non-musicians, musicians were faster and more frequent users of MD strategies, which incorporate both pitch height and pitch direction information- an advantageous strategy that promotes greater differentiation between tone categories.

While learning is important, generalization of the learned material is also important, especially in the case of speech as it rarely occurs in the same context. Different talkers with variable speaking characteristics such as rate of speech, average pitch, etc., all create unique contexts in which speech must be understood. Therefore, in addition to during the five blocks of learning, we examined accuracies, strategies, and perceptual selectivity during a generalization block in which participants were required to categorize the four Mandarin tones in the context of a single, new speaker and received no feedback. Musicians showed an accuracy advantage that was supported by enhancements in both decisional strategies (larger number of MD users) and perceptual selectivity (smaller perceptual variance along pitch height and pitch direction dimensions). A large literature suggests that non-native speech sound training which implements highly variable training stimuli is more conducive than low variable training stimuli to successfully generalizing learned speech sounds to new contexts (see Bradlow, 2008; Perrachione et al., 2011). Importantly, prior research has manipulated the training paradigm in order to produce successful generalization. The current results build off of this literature and suggest there may also be individual differences (such as music training) involved in how successful a participant is in generalizing learned non-native speech sounds to novel contexts. Future research should investigate how and which individual differences lead to successful generalization of learned non-native speech sounds.

The burgeouning literature on the cross-domain plasticity induced by long-term music training has led several researchers to propose music training as a clinical training tool. Our current findings hold promise for using long-term music training as a method to help clinical populations that demonstrate greater auditory–perceptual variability (Hornickel and Kraus, 2013) and learning-related difficulties. However, on a cautionary note, several questions and criticisms should be addressed before pursuing more clinical goals. For example, first, it is unclear whether the cognitive and perceptual advantages reflect an effect of long-term music training, or a general predisposition that drives individuals toward music training. A recent longitudinal study suggests the former (Kraus et al., 2014). Using a longitudinal design, children from the Los Angeles area were randomly assigned to either defer music involvement for a year and receive only 1 year of music lessons, or begin music instruction immediately and receive a total of 2 years of music training. By the end of the 2-year training, the second group, which had received 2 years of music training, showed stronger neurophysiological distinctions of /ba/ versus /ga/ sounds, while the first group did not. In addition, within the second group, number of hours spent practicing over the 2-year training period positively correlated with improvement in neural differentiation (Kraus et al., 2014). However, there were several limitations that prevent strong inferences from being drawn. For instance, an active control group against which they could compare the gains in the 2-year music group was not included. In addition, there were several issues regarding analyses of the data, and no behavioral data were presented (Evans et al., 2014). Next, we need to evaluate the specificity of the musician advantage. Pitch and changes in pitch are clearly important attributes of music. Whether cognitive and perceptual advantages percolate to other attributes of sound such as loudness and duration need to be addressed in future studies. Lastly, in the current study we use a definition of ‘musician’ that is derived from the larger existing literature; however, this definition is admittedly narrow (see Levitin, 2012 for example), as is the definition of a ‘non-musician.’ In addition, a larger sample size, allowing the examination of music training to be a continuous variable, and a well-established performance-based measure would prove useful.

### Future Directions

There are many available future directions. One is to more broadly explore the extent of the observed musician cognitive advantage in speech learning. For instance, cognitive tasks that show musician advantages are frontally mediated cognitive tasks that test executive function (Bialystok and DePape, 2009), working memory (Parbery-Clark et al., 2009; Pallesen et al., 2010; George and Coch, 2011; Kraus et al., 2012; Strait et al., 2013), and switching (Hanna-Pladdy and MacKay, 2011). Musicians also show increased gray matter volume in the dorsolateral prefrontal cortex (Bermudez et al., 2009). Given that musicians show frontally mediated advantages, it is possible these complexs frontally mediated rule-based strategies drive cross-domain auditory plasticity, especially given the task-dependent nature of activation in the human auditory cortex (Ohl and Scheich, 2005). Notably, when construed within a dual-learning systems perspective, a rule-based learning advantage may not transfer to all learning conditions. Within the dual-learning systems framework, a *reflective* system, which uses executive attention and working memory, is *competitive* with the *reflexive* system, which relies on dopamine-mediated reward signals in the striatum (Ashby and Maddox, 2005, 2011; Maddox et al., 2013; Maddox and Chandrasekaran, 2014). Since these two systems are competitive, if the musician advantages in cross-domain plasticity are driven purely by the frontally mediated cognitive advantages, musicians should perform worse on auditory tasks that require the reflexive, striatally mediated, system than on auditory tasks that require the reflective system. Thus a robust theoretical framework may help establish the limits of neuroplasticity related to music training.

A second future direction is to investigate whether different music-training environments provide different cognitive or perceptual benefits related to non-native speech learning. In the present study, we used musicians who have at least 10 years of formal group or private training. It is possible that musicians with less training, those who play instruments from different instrument families, those who are self-taught, or those who play instruments that use non-Western tonality will show different learning patterns compared to the musicians in this study. For instance, many non-Western styles of music use tonalities that distinguish between smaller differences in pitch than Western music. This may result in greater demands on the perceptual system, and consequently lead to a non-Western trained musician advantage over Western-trained musicians in learning non-native speech sounds due to the increased sensitivity to smaller pitch differences. Lastly, research suggests that non-human species are capable of differentiating between different types of pitch movements – a skill trained during music learning and used in non-native speech learning (Ohl et al., 2001; Brosch et al., 2004). As suggested by Patel (2014), animal models may provide valuable insight into how specific aspects of music training (i.e., pitch movements) may influence species-specific language components such as vocalizations, and thus clarify how music training may affect speech learning.

## Conclusion

Using rigorous computational modeling, we extended prior research by showing that the musician accuracy advantage relative to non-musicians observed in prior studies can be attributed to both cognitive advantages, as evidenced by earlier and more frequent use of the optimal MD strategy; and perceptual advantages, as evidenced by smaller perceptual noise along both the pitch height and pitch direction dimensions. In addition, musicians and non-musicians who used MD strategies by the end of training showed no differences in accuracy and working memory scores. Contrastingly, participants who used MD strategies by the end of training showed higher accuracy rates and working memory scores than those who used UD or RR strategies. These results suggest a cognitive mechanism for the musician accuracy advantage. Specifically, the use of MD strategies faster and more often relative to non-musicians. In the generalization block, where stimuli were presented by a new talker, and no feedback was given, more musicians used the optimal strategy and obtained a higher accuracy relative to non-musicians. At the perceptual level, our modeling revealed that musicians’ perception of the stimuli is more veridical, especially along the normally underweighted pitch direction dimension. This pattern extended to novel stimuli during a generalization phase. These results provide further evidence for cross-domain auditory plasticity due to music training.

## Conflict of Interest Statement

The authors declare that the research was conducted in the absence of any commercial or financial relationships that could be construed as a potential conflict of interest.
